# Higher blood lipid levels after the transition to menopause in two forager-horticulturalist populations

**DOI:** 10.1093/emph/eoaf020

**Published:** 2025-07-20

**Authors:** M J Getz, J E Aronoff, C L Jenkins, S Ghafoor, J Vazquez, N T Appel, M Gatz, D K Cummings, P L Hooper, B Beheim, K H Buetow, C E Finch, G S Thomas, J Stieglitz, M Gurven, H Kaplan, B C Trumble

**Affiliations:** Center for Evolution and Medicine, Arizona State University, Life Sciences C, 427 East Tyler Mall, Tempe, AZ 85281, USA; Institute of Human Origins, Arizona State University, Walton Center for Planetary Health, 777 East University Drive, Second Floor #200L1, Tempe, AZ 85287, USA; School of Human Evolution and Social Change, Arizona State University, 900 Cady Mall, Tempe, AZ 85281, USA; Center for Evolution and Medicine, Arizona State University, Life Sciences C, 427 East Tyler Mall, Tempe, AZ 85281, USA; Center for Evolution and Medicine, Arizona State University, Life Sciences C, 427 East Tyler Mall, Tempe, AZ 85281, USA; Center for Evolution and Medicine, Arizona State University, Life Sciences C, 427 East Tyler Mall, Tempe, AZ 85281, USA; Center for Evolution and Medicine, Arizona State University, Life Sciences C, 427 East Tyler Mall, Tempe, AZ 85281, USA; Center for Evolution and Medicine, Arizona State University, Life Sciences C, 427 East Tyler Mall, Tempe, AZ 85281, USA; Center for Economic and Social Research, University of Southern California, 635 Downey Way, Los Angeles, CA 90089, USA; Economic Science Institute, Chapman University, 1 University Drive, Orange, CA 92866, USA; Economic Science Institute, Chapman University, 1 University Drive, Orange, CA 92866, USA; Department of Human Behavior, Max Planck Institute for Evolutionary Anthropology, Deutscher Platz 604103, Leipzig, Germany; Center for Evolution and Medicine, Arizona State University, Life Sciences C, 427 East Tyler Mall, Tempe, AZ 85281, USA; Center for Economic and Social Research, University of Southern California, 635 Downey Way, Los Angeles, CA 90089, USA; MemorialCare Heart & Vascular Institute, MemorialCare Health System, 9920 Talbert Ave, Fountain Valley, CA 92708, USA; Division of Cardiology, University of California Irvine, 333 City Blvd West, Suite 400, Orange, CA 92868, USA; Institute for Advanced Study, Toulouse School of Economics, 1, esplanade de l'Université, 31080 Toulouse Cedex 06, France; Integrative Anthropological Sciences, University of California Santa Barbara, Humanities and Social Sciences Building (HSSB) 2001, Santa Barbara, CA 93106, USA; Economic Science Institute, Chapman University, 1 University Drive, Orange, CA 92866, USA; Center for Evolution and Medicine, Arizona State University, Life Sciences C, 427 East Tyler Mall, Tempe, AZ 85281, USA; Institute of Human Origins, Arizona State University, Walton Center for Planetary Health, 777 East University Drive, Second Floor #200L1, Tempe, AZ 85287, USA; School of Human Evolution and Social Change, Arizona State University, 900 Cady Mall, Tempe, AZ 85281, USA

**Keywords:** menopause, lipids, cardiovascular disease, forager-horticulturalists, aging

## Abstract

**Background:**

Reproduction affects health and longevity among females across the life course. While significant focus has been devoted to the role of menarche, menopause remains understudied. Most menopause research is conducted in industrialized populations, where the risk of cardiovascular diseases increases progressively during the menopausal transition.

**Methodology:**

We worked with the Tsimane, Indigenous Bolivian forager-farmers with physically active lifestyles, and the Moseten, genetically and culturally related horticulturalists experiencing greater market integration. We assessed relationships between menopause status and lipid biomarkers (HDL, LDL, non-HDL, total cholesterol, triglycerides, and apolipoprotein-B). Using linear mixed-effects models, in an all-age sample of n = 1,121 women (15–92 years) we found positive associations between menopausal status and most lipid levels.

**Results:**

Menopause was associated with 5.0% higher total cholesterol (b = 7.038 mg/dL, *P* = .001), 9.4% higher LDL (b = 5.147 mg/dL, *P* = .017), 5.9% higher non-HDL cholesterol (b = 8.071 mg/dL, *P* < .001), 11.3% higher triglycerides (b = 19.119 mg/dL, *P* < .001), and 1.5% higher apolipoprotein-B (b = 0.248 mg/dL, *P* = .001), controlling for age, body mass index (BMI), year of data collection, and population. In contrast, HDL did not vary with menopause status.

**Conclusions:**

After controlling for age, BMI, and year of data collection, post-menopausal lipid profiles among the Tsimane across six biomarkers are 2–7 times lower than those documented in U.S./U.K. populations. These results support existing literature that documents distinct shifts in lipid profiles during and after the menopause transition in industrialized populations. Further, our results suggest lipids increase post-menopause similarly to those of industrialized populations, despite the differential diet, physical activity, fertility, and hormone exposure in industrialized environments.

**Lay Summary:**

Menopause is a relatively rare life history trait primarily studied in industrial populations. We examined relationships between menopause and cardiovascular disease risk biomarkers in two forager-horticulturalist populations. We found positive associations between menopause and total cholesterol, HDL, LDL, non-HDL, triglycerides, and apolipoprotein-B, suggesting lipid increases post-menopause are a human universal.

## BACKGROUND

Humans are among the few species with long post-reproductive lifespans [1–3]. As much as one-third of women’s lifespan is post-reproductive period. While a handful of species survive past reproduction in captivity, it is rare for most species to switch from direct to indirect reproductive effort in the wild [2, 4, 5]. Nonetheless, there are still a number of health consequences associated with the transition to menopause. Rapid increases in cardiovascular disease, depression, osteoporosis, and breast cancer have been observed in post-menopausal women in high-income countries [6–8]. Cardiovascular disease in particular is a concern; rates of cardiovascular disease increase dramatically post-menopause, and cardiovascular disease is currently the leading cause of death for women worldwide [9, 10]. Given this increased risk of cardiovascular disease post-menopause, this study will assess if the menopausal increases in lipid levels that have been reported in sedentary industrialized populations are generalizable to physically active subsistence populations with low levels of cardiovascular disease.

The force of selection is expected to decline post-menopause, since direct reproduction has ceased [11]. Natural selection optimizes reproduction, not health or longevity (unless those benefit reproduction). As such, the force of selection declines after direct reproduction ends, though potential inclusive fitness benefits of a long post-reproductive lifespan may prevent a complete decline of this force to zero [5, 12]. It is thus expected that there will be increases in a number of health conditions, including an increase in cardiovascular disease risk, following the transition to menopause.

Scholars debate both the evolutionary timing and the selection pressures that led to a long post-reproductive life in humans [4]. Prominent theories on the evolution of a long post-reproductive lifespan include the Grandmother Hypothesis, where post-reproductive longevity supports inclusive fitness of kin through provisioning of offspring and grandchildren [5], and the Mother Hypothesis, which argues post-reproductive lifespans allow mothers to raise their last children to self-sufficiency [13]. Embodied Capital Theory argues that a long post-reproductive lifespan exists to facilitate intergenerational resource transfers between older individuals who produce surplus calories and juveniles unable to meet their caloric needs while learning complex skills like hunting [2, 14, 15].

**Figure 1:**
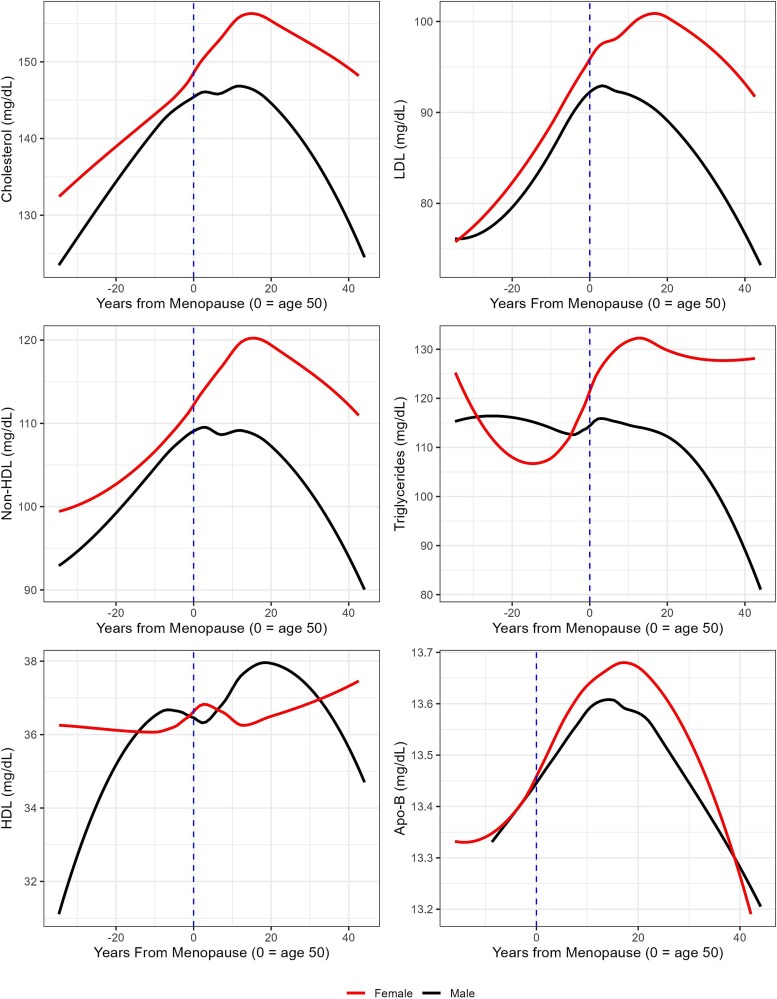
Six graphs showing trends for male and female cholesterol, HDL, non-HDL, triglycerides, LDL, and Apo-B by age/years from menopause.

Regardless of its evolutionary origins, a variety of health changes have been associated with the menopause transition, including increased levels of low density lipoprotein (LDL) cholesterol, non-high density lipoprotein (non-HDL) cholesterol, varied levels of triglycerides and apolipoprotein-B (Apo-B), changes in LDL and HDL cholesterol particle size, cognitive declines, and decreasing levels of estradiol [16–21]. However, the biological mechanisms underlying these changes are contested [22]. Post-menopausal declines in estrogen may reduce its cardioprotective effects—such as improved endothelial function, increased arterial elasticity, and reduced inflammation—thereby elevating cardiovascular disease risk markers [23–25]. These impacts may differ between industrialized and non-industrial, high-fertility populations due to differences in hormone exposures (e.g. patterns of pregnancy and lactation, hormonal contraceptive use). However, it is unclear if hormonal and metabolic changes occur as a consequence of aging or as a direct result of the menopause transition [22]. In our study populations, we observe markedly distinct cardiovascular risk marker profiles for women after menopause and men after age 50 (see Fig. 1 for population-level trends in these profiles by sex).

Pregnancy and lactation are energetically costly [26], and increases in cardiovascular disease risk post-menopause have been attributed to increased caloric availability after the end of reproduction. For example, Tsimane women have 9.1 children on average, resulting in a major energetic cost throughout the reproductive lifespan [27]. After menopause, we see increases in body mass as these costs disappear [27, 28]. Greater net caloric availability may also contribute to an increasingly atherosclerotic lipid profile alongside declining estrogen levels. However, these results conflict with other research in high-fertility environments like Papua New Guinea that have identified maternal depletion, or lower body mass index (BMI) associated with increased fertility [29].

Most data on menopause to date have been collected from historically white, industrialized populations [30]. Because of this sample bias, much of our knowledge on the health effects of menopause and their variation are based on poorly generalizable models. These rely on data from individuals with meaningfully different hormone exposures and physical activity levels (PALs) than subsistence-oriented, food-limited high-fertility environments [30, 31]. Working with two Bolivian horticultural populations—the Tsimane and Moseten—we address this knowledge gap to examine associations between menopause and cardiovascular disease risk factors in a less industrialized, traditional high-fertility environment [27, 28, 32, 33]. The Tsimane have shown the lowest levels of coronary arterial calcium recorded [33], with low levels of hypertension and other cardiac issues [34–36]. This positions the Tsimane as a compelling comparator population, given the high burden of cardiovascular disease in industrialized populations. If similar associations between cardiovascular risk biomarkers and menopause are observed in the Tsimane and Moseten as those seen in industrialized populations, this suggests that menopause-related shifts in cardiovascular profiles are a human universal, despite differences in population-level cardiovascular risks.

The Tsimane are an Indigenous Bolivian group numbering roughly 17,000. Residing largely in communities along the Maniqui River, a tributary of the Amazon, the Tsimane live a physically active subsistence lifestyle [37]. Common subsistence practices include hunting, fishing, foraging, and farming with hand tools. 24-hour PALs among Tsimane men and women are 2.02–2.15 and 1.73–1.85, respectively [38]. This high level of activity contrasts with those seen in industrialized populations—U.S. adults have an estimated mean PAL of 1.63 [39]. Communities have limited access to treated water, sanitation services, pharmaceuticals, or electricity [40–43]. The Moseten are linguistically, genetically, and culturally related to the Tsimane, but are horticulturalists experiencing greater levels of market integration [44]. Estimated at around 3,000 individuals, the Moseten are generally more acculturated into Bolivian society and have greater access to electricity, market foods, and medications. The Moseten have been shown to consume diets substantially higher in refined sugars and oils than the Tsimane [45], display lower PALs [35], and have slightly lower fertility rates at around six children per woman. While more market-integrated, the Moseten still live in rural communities and participate in labor-intensive agriculture [35, 44, 45].

Given the decline in the force of selection after direct reproduction has ceased and eliminated energetic costs of direct reproduction, we expect to see higher lipid levels in the Tsimane and Moseten post-menopause. Extra calories reallocated from reproduction could lead to higher lipid production. These elevated lipids would be produced in selection’s shadow, leading to higher levels of cardiovascular disease among post-menopausal women. LDL cholesterol, HDL cholesterol, non-HDL cholesterol, total cholesterol, triglycerides, and Apo-B are associated with cardiovascular health [46–49]. We collected cross-sectional and longitudinal data from 2004 to 2022 on these biomarkers and menopause status as a part of the Tsimane Health and Life History Project. We aim to test the associations between these biomarkers and menopause status, providing an opportunity to assess the relationship between menopause status and cardiovascular disease risk in two non-industrialized populations. To the best of our knowledge, this is the largest sample size of lipid-menopause measurements in any Indigenous population.

These two populations provide an opportunity to investigate potential changes associated with menopause in a population undergoing market transition and another living a more traditionally active lifestyle. We expect the Tsimane lipids to be lower than U.S./U.K. comparator populations given their high fertility levels, relatively high PALs, and physically active subsistence strategies [37, 38]. While the Moseten maintain relatively high fertility and PALs compared to U.S./U.K. populations, we predict their cardiovascular risk markers will be more intermediate between industrialized populations and the Tsimane as they have undergone greater levels of market integration. For example, the Moseten display significantly higher levels of obesity, diabetes, and hypertension than the Tsimane. Moseten metabolic risk biomarkers remain significantly lower than comparably aged U.S. adults [35]. In our analysis, we aim to provide some of the first results of their kind on the association between menopause and cardiovascular disease risk markers, as well as the global variation of cardiovascular disease risk markers across the menopause transition.

## METHODOLOGY

### Sample

Our sample was comprised of 2,216 serum specimens for lipid analyses from 1,121 women ages 15–92, including 850 Tsimane and 271 Moseten. Our sampling strategy included all individuals over the age of 45 and age-stratified random sampling of individuals younger than 45. We also included male Tsimane (n = 1,768, aged 15–94) and Moseten (n = 421, aged 22–89) as a comparator group (Fig. 1).

### Demographic interviews

Demographic interviews were conducted in the Tsimane language with a bilingual Tsimane translator. Among the Moseten, interviews were conducted in Spanish. Years of birth were assigned and cross-referenced using known ages from written records, photo comparisons of individuals with known ages, relative age rankings by multiple informants, and dated events [50]. Where possible, estimated ages were compared against Catholic mission birth records dating back to 1952 [50]. Each method produced a roughly independent age estimate, and the average of these estimates was used if all fell within a three-year age range, unless one method was considered superior to others (see Gurven et al., 2007).

### Reproductive status

Age at menopause was assessed using two methods: self-reported menopause status with age and yearly medical visits asking date of last menstrual cycle. In routine health screenings approximately every 16 months between 2002 and 2022, women were asked about the total number of live births they had ever had, live births since their last health screening, total number of pregnancies (including miscarriages and other losses), total number of pregnancies since their last screening, and the date of the last menstruation. Among the Tsimane and Moseten there is no taboo around discussing child death or fetal loss. However, early fetal loss is likely under-detected and underreported. There is no word for menopause in Tsimane, and menopause status was back-calculated from the date of a participant’s last menstrual cycle [28]. A subset of 459 women in our sample had detailed reproductive histories originally collected in 2002–2005 and updated through 2022. Women over age 50 who lacked data on age at menopause were assigned post-menopause status at age 50. Individuals assigned menopause status comprised 35.6% of our post-menopause observations. Nine individuals self-reported undergoing menopause at age 38 and 39. An estimated less than 1% of women undergo menopause before the age of 40 in industrialized populations [51]. Given this low estimate, the nine individuals were removed from our post-menopause sample.

### Lipid biomarker collection and assay procedures

Fasting morning blood (n = 2,216 specimens) was collected and separated into serum before being frozen in liquid nitrogen and transferred to Arizona State University on dry ice before storage at -80C. A commercial immunoassay was used to measure Apo-B (R&D Systems, Minneapolis, Minnesota). Fasting lipids (HDL, LDL, total cholesterol, triglycerides) were measured from serum samples using a Stat Fax 1908 (Awareness Technology, Palm City, Florida) in the Tsimane Health and Life History Project’s laboratory in San Borja, Beni, Bolivia [33]. Non-HDL cholesterol was calculated by subtracting HDL from total cholesterol. Not all individuals had complete lipid data collected for all measures due to lack of availability of some reagents in Bolivia at the time of analyses; Apo-B was only run on a subset of individuals over age 40 (n = 357).

### Anthropometric measure collection procedures

BMI was calculated using weight (kg) divided by height (m) squared. Standing height was measured without shoes to the nearest millimeter with a SECA 213 mobile stadiometer (Seca, Hamburg, Germany). Weight was collected using a Tanita BC-1500 scale (Tanita, Tokyo, Japan).

### Statistical methods

All main models were run in STATA 17.0, and sensitivity analyses and cohort effects were run in R version 4.4.2. Logistic regressions for clinically high values were run in STATA 17.0. We performed mixed-effects linear regression models using menopause status and LDL cholesterol, HDL cholesterol, non-HDL cholesterol, total cholesterol, triglycerides, and Apo-B, controlling for age, BMI, year of data collection, and population and centered on age, BMI, and year of data collection. Individuals were modeled as random effects; 50.6% of individuals in the sample provided longitudinal data (n_observations_ = 1,122). Variance inflation factors (VIFs) were run for all variables included in the model to account for potential collinearity.

### Ethics

Informed consent was collected at three levels: from the individuals in the study, each community where data were collected, and the governing bodies of the Tsimane (Gran Consejo Tsimane) and Moseten (Organización del Pueblo Indígena Mosetén). All study protocols were approved by the Institutional Review Boards at the University of California Santa Barbara (#3-21-0652), the University of New Mexico (#07–157), and the Universidad Mayor de San Simón (Cochabamba, Bolivia).

## RESULTS

Menopause was associated with higher total cholesterol, LDL cholesterol, non-HDL cholesterol, triglycerides, and Apo-B among the Tsimane and Moseten, controlling for age, BMI, year, and population (see Table 1).

**Table 1:** Review of study characteristics, mean/median biomarker values, and percent difference by menopause status.

				**Pre-menopause**					**Post-menopause**						**Mean percent change**				
**Citation**	**Population**	**Age**	**n**	**TC**	**LDL**	**HDL**	**Non-HDL**	**TG**	**n**	**TC**	**LDL**	**HDL**	**Non-HDL**	**TG**	**TC**	**LDL**	**HDL**	**Non-HDL**	**TG**
Anagnostis et al. (2015) [52]	U.K.	32.4†, 56†	515	*157*	*116.5*	*63.4*	*94.8*	*58.5*	518	*216.2*	*118.4*	*61.5*	*154.3*	*90.3*	37.7%^***^	1.6%^***^	3.0%^**^	62.8%^***^	54.5%^***^
Derby et al. (2009) [53]	SWAN/U.S.	46††	2659	196.7	116.3	57.7		100.2	1058	205.2	123.1	57.7		106.4	4.20%	5.60%	0.0%^***^		6.20%
Stevenson et al. (1993) [54]	U.K.	18–70	395	190.6	108.3	70		57.6	147	216.7	137.7	65		64.7	13.7%^***^	27.2%^***^	−7.2%^***^		12.3%^**^
Woodard et al. (2011) [55]	SWAN/U.S.	50††	316		103	58.2		91	224		139	55.8		124		35.0%^***^	4.10%		36.3%^*^
Bermingham et al. (2022) [56]	U.K.		366	178.3	100.5		113.7	83.2	206	209.9	125.7		137.4	96.5	17.7%^*^	25.00%		20.80%	16.0%^***^
**U.S./U.K. average % change**															18.40%	18.90%	0.00%	41.80%	25.10%
	Moseten	33–89	120	153	103	35.6	117.4	153	292	169	108	35.1	133.9	165	7.6%^***^	4.9%^*^	−1.40%	14.1%^***^	7.8%^***^
	Tsimane	15–92	826	141	87.3	36.3	104.7	106	978	148	95.5	37.1	110.9	118	5.0%^***^	9.4%^*^	2.20%	5.9%^***^	11.3%^***^

†mean age for pre-menopause, post-menopause

††mean age for total sample

^*^ indicates *P* ≤ .05, ^**^ indicates *P* ≤ .01, ^***^ indicates *P* ≤ .001

All values expressed in mg/dL. Results include all mean or median (indicated by italics) values reported from original research articles. For publications where pre- and post-menopause were divided into stages, early perimenopause was considered pre-menopause, and late perimenopause was considered post-menopause (see Woodard et al., 2011).

We found positive associations between total cholesterol and menopause status (beta = 7.038 mg/dL, CI = 2.710–11.376 mg/dL, *P* = .001, see Table 2). LDL cholesterol was also positively associated with menopause (beta = 5.147 mg/dL, CI = 0.913–9.381 mg/dL, *P* = .017, see Table 2). Overall, after controlling for age, BMI, and year of data collection, total cholesterol was 5.0% higher in post-menopausal women, while LDL cholesterol was 9.4% higher after menopause. Non-HDL cholesterol (beta = 8.071 mg/dL, CI = 3.778–12.364, *P* < .001) and triglycerides (beta = 19.119 mg/dL, CI = 11.468–26.770 mg/dL, *P* < .001) were both positively associated with menopause status (see Table 2). Non-HDL cholesterol levels were 5.9% higher post-menopause, and triglycerides were 11.3% higher post-menopause compared to pre-menopause values. Apo-B was also positively associated with menopause (beta = 0.248, CI = 0.107–0.390, *P* = .001) (Table 2) and was 1.5% higher post-menopause compared to pre-menopause. We found no significant association between menopause and HDL cholesterol (see Table 2). VIFs for each variable in each model—total cholesterol (2.29, 2.50, 1.14, 1.40, and 1.63 for menopause, age, BMI, population, and year of data collection, respectively), LDL (2.25, 2.44, 1.14, 1.40, and 1.61), non-HDL (2.25, 2.45, 1.14, 1.40, and 1.61), triglycerides (2.30, 2.50, 1.14, 1.40, and 1.63), Apo-B (1.72, 1.74, 1.02, 1.07, and 1.05), and HDL (2.25, 2.45, 1.14, 1.40, and 1.61)—were all ≤2.5. In addition to assessing collinearity between age and menopause status, our model included both age and year of data collection to control for cohort effects due to increased market integration and lifestyle change.

**Table 2:** Beta values for separate mixed-effects regression models for total cholesterol, LDL, HDL, triglycerides, non-HDL, and apo-B in Tsimane and Moseten women.

	**Total Cholesterol (n = 1112, n observations = 2207)**			**LDL (n = 1047, n observations = 2033)**			**HDL (n = 1047, n observations = 2033)**			**Triglycerides (n = 1111, n observations = 2204)**			**Non-HDL (n = 1047, n observations = 2035)**			**Apo-B (n = 279, n observations = 358)**		
	**β**	**95% CI**	**p**	**β**	**95% CI**	**p**	**β**	**95% CI**	**p**	**β**	**95% CI**	**p**	**β**	**95% CI**	**p**	**β**	**95% CI**	**p**
**Menopause**	7.038	2.701, 11.376	0.001	5.147	0.913, 9.381	0.017	−0.041	−1.098, 1.016	0.94	19.119	11.468, 26.770	<0.001	8.071	3.778, 12.364	<0.001	0.248	0.107, 0.390	0.001
**Age**	−0.016	−0.191, 0.156	0.858	0.083	−0.087, 0.254	0.337	0.016	−0.026, 0.058	0.449	−0.156	−0.469, 0.158	0.33	−0.063	−0.238, 0.112	0.483	0.001	−0.005, 0.007	0.782
**BMI**	1.173	0.802, 1.544	<0.001	1.017	0.661, 1.373	<0.001	−0.199	−0.287, −0.112	<0.001	3.603	2.931, 4.275	<0.001	1.345	0.977, 1.712	<0.001	0.012	0.001, 0.023	0.034
**Moseten**	9.092	4.478, 13.705	<0.001	4.943	0.565, 9.320	0.027	−1.003	−2.082, 0.076	0.069	35.593	27.258, 43.928	<0.001	9.516	5.00, 14.028	<0.001	−0.207	−0.388, −0.025	0.026
**Year**	1.043	0.668, 1.418	<0.001	0.991	0.624, 1.358	<0.001	0.029	−0.063, 0.121	0.536	−0.304	−0.961, 0.353	0.364	1.099	0.668, 1.418	<0.001	0.033	0.001, 0.064	0.045
**Constant**	143.455	140.358, 146.551	<0.001	90.201	87.185, 93.217	<0.001	36.67	87.185, 93.217	0.85	104.16	98.608, 109.711	0.343	106.018	102.925, 109.112	<0.001	13.332	13.226, 13.437	0.108

β = the increase observed in the dependent variable (mg/dL) for each one unit increase in the dependent variable

### Population differences

**Figure 2:**
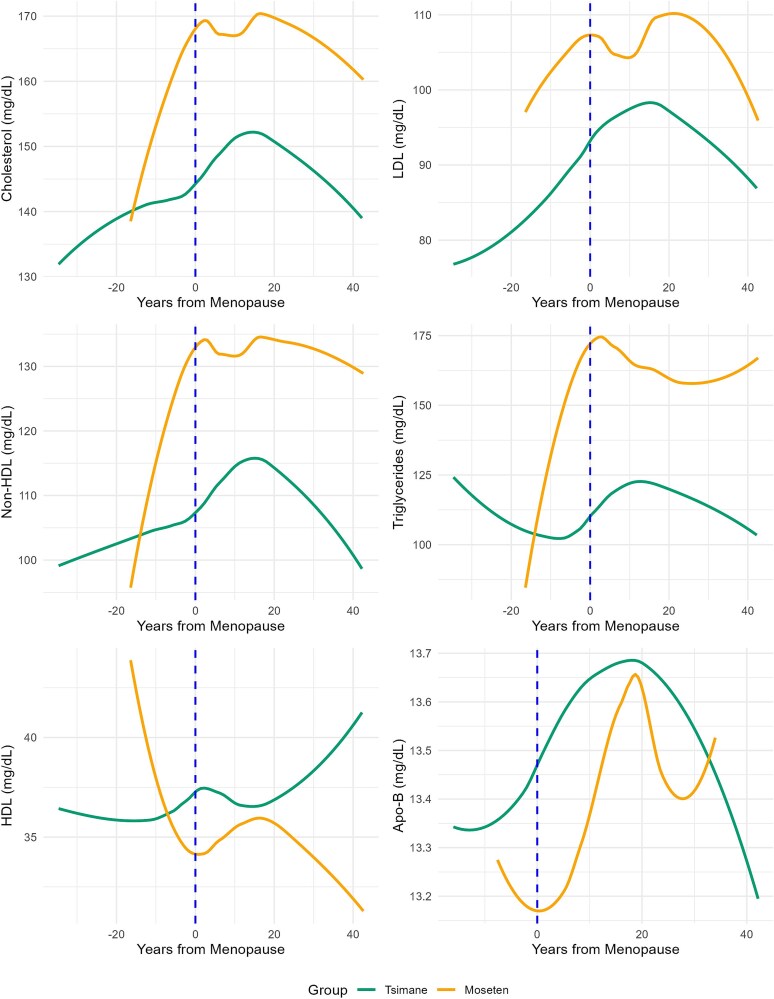
Six graphs showing trends for female Tsimane and Moseten cholesterol, HDL, non-HDL, triglycerides, LDL, and Apo-B by years from menopause.

Most lipid levels were higher among Moseten than Tsimane women, including total cholesterol (beta = 9.092 mg/dL, CI = 4.478–13.705, *P* < .001), non-HDL (beta = 9.516 mg/dL, CI = 5.00–14.028, *P* < .001), and triglycerides (beta = 35.593, CI = 27.258–43.928, *P* < .001) (see Table 2). Total cholesterol levels, after all controls, were 7.6% higher among post-menopausal Moseten women, while non-HDL and triglycerides were 14.1% and 7.8% higher for this group, respectively (Table 1). The Moseten did not significantly differ from Tsimane in LDL, HDL, or Apo-B (Fig. 2).

### Sensitivity analysis

In our sensitivity analysis comparing self-report versus assigned menopause women there were no significant differences for LDL, total cholesterol, or Apo-B. HDL (beta = 1.762, *P* < .001) and triglycerides (beta = −0.145, *P* < .001) were significant, but these differences were minimal (4.9% and −0.1% of total sample post-menopause means, respectively). The mean age at menopause of participants who self-reported menopause status was 48.6 years (SD = 4.48), and mean age of participants assigned menopause status was 61.3. Largely, women assigned menopause status were elderly individuals who could not recall their age at menopause, nor time since their last menses. For details, see SI and Tables S1, S2, and S3.

### Clinically high values

**Figure 3:**
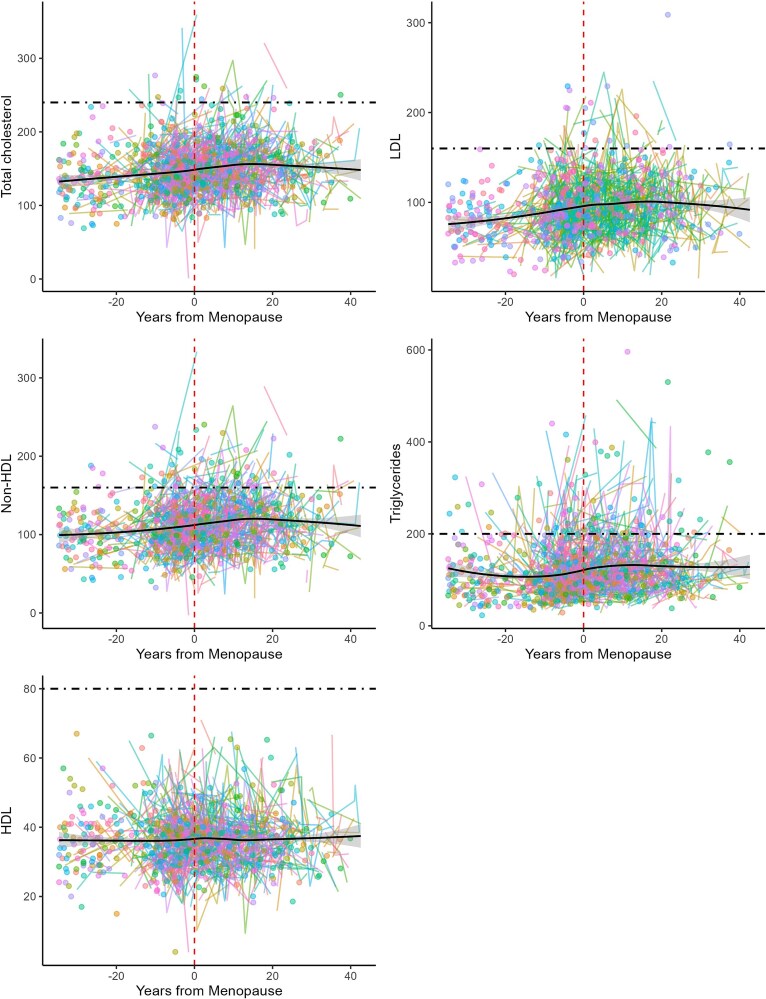
Five spaghetti plots showing individual values for total cholesterol, HDL, non-HDL, triglycerides, and LDL by years from menopause.

Menopause status was associated with increased risk of clinically high levels of non-HDL (OR = 2.33, *P* = .003), total cholesterol (OR = 3.00, *P* = .003), and triglycerides (OR = 2.52, *P* < .001) (Fig. 3). Menopause status did not significantly predict high LDL or HDL cholesterol (see SI for methods).

## DISCUSSION

In this study, we investigated the association between menopause and cardiovascular disease risk biomarkers (HDL, LDL, total cholesterol, non-HDL cholesterol, triglycerides, and Apo-B) in a non-industrial, high-fertility population with the lowest levels of coronary artery calcium ever reported [33, 35]. Our results indicate that among the Tsimane and Moseten, total cholesterol, LDL, non-HDL cholesterol, triglycerides, and Apo-B are significantly higher among post-menopausal women. Triglycerides show the largest increase among the Tsimane post-menopause (an 11.3% increase in post- vs pre-menopause beta values when controlling for age, BMI, and year of data collection), while non-HDL cholesterol shows the largest increase among the Moseten (14.1% in our model with all controls). Additionally, our results indicate that being post-menopausal increases an individual’s odds of having high non-HDL cholesterol at 2.3x that of their pre-menopause counterparts, increases the odds of high total cholesterol by 3.0x, and increases the odds of high triglycerides at 2.5x that of the pre-menopausal rate. We did not find significant associations between menopause status and HDL cholesterol.

Our results are consistent with earlier longitudinal and cross-sectional work that documents significant increases in total cholesterol, LDL cholesterol, non-HDL cholesterol, and triglycerides during and after the menopause transition in high-income settings [21, 57–62] (see Fig. 3 for lipid changes among our longitudinal participants). However, in some cases, increases among the Tsimane appear to be reduced compared to industrialized populations. Compared to existing studies, post-menopause total cholesterol levels from the U.S. Study of Women’s Health Across the Nation (SWAN) are roughly comparable to Tsimane women [59]. However, studies among white women based in the U.K. have shown total cholesterol levels ranging from 13.7 to 37.7% higher among post-menopausal women, roughly three to seven times the magnitude of observed differences in the Tsimane after controlling for age, year of data collection, and BMI [58, 61, 63]. While post-menopause Tsimane and Moseten women do show significantly higher total cholesterol levels than pre-menopause participants, these increases are considerably lower—both relatively and absolutely—than some industrialized populations.

LDL cholesterol levels among post-menopausal Tsimane women were roughly 9% higher than their pre-menopausal counterparts (after controlling for age, year of data collection, and BMI). Using the same controls, the Moseten saw a 4.9% increase in post-menopausal women, similar to what has been described in U.S. populations [59]. There is a good deal of variation in the literature, with increases ranging from 1.6% to 35.0% in high-income settings [58, 61–63].

Non-HDL cholesterol increased 5.9% and 14.1% among the Tsimane and Moseten after controlling for age, year of data collection, and BMI, while high-income studies report increases ranging from 20.8% to 62.8% in industrialized populations [58, 61]. These changes are 3.5–10.6 times greater than those observed in the Tsimane and Moseten. For LDL, non-HDL, and total cholesterol, Fig. 2 displays slight decreases in mean levels roughly 20 years after menopause.

Triglyceride levels also show marked variation across populations. The Tsimane experience an 11.3% increase, similar to white women in the U.K. [61] and higher than that of the Moseten and some SWAN data, even after controlling for year of data collection, BMI, and age [59]. However, other studies using SWAN and U.K. data have shown 6.2%–54.5% increases among post-menopausal women [58, 62, 63].

We found a modest but significant 1.5% increase in Apo-B beta values among the post-menopausal Tsimane after applying all controls. This is consistent with existing studies that display significant increases in Apo-B associated with the menopause transition, though mean values for cohorts were not available for direct comparison [16, 21, 64].

For all markers collected, we see substantial variation in both absolute and relative increases across populations. We did find significant differences between the U.S./U.K. and Moseten and Tsimane pre-to-post-menopause baseline values and percent change for total cholesterol and HDL (see Table S4). Longitudinal results from SWAN report that higher pre-menopause weight is associated with larger increases in lipids post-menopause, particularly among white U.S. women [59]. However, the mechanisms underlying this variation are largely unclear. Similarly, we see pronounced variation in percent changes between the Tsimane and Moseten, with LDL and triglyceride levels showing greater increases among the Tsimane and higher non-HDL cholesterol and total cholesterol increases among the Moseten. Some of these results, as well as the differences between Tsimane and U.S./U.K. changes, could be partially attributable to higher PALs and significantly lower amounts of oil, sugar, and fat in Tsimane diets [35, 38, 45]. However, these diet and activity differences are unlikely to account for greater LDL and triglyceride increases among the Tsimane when compared to the Moseten.

Overall levels of total cholesterol, LDL, non-HDL cholesterol, triglycerides, and Apo-B appear to rise for the first 10–20 years following menopause (Fig. 3). Menopause eliminates the energetic burdens of fertility, freeing calories previously allocated for reproduction. Without a corresponding energy cost, this available energy is carried through the blood in the form of glucose and lipids, including HDL, total cholesterol, and triglycerides. The Tsimane experience low levels of cardiovascular disease relative to industrialized populations [33], high levels of fertility, and demonstrate increases in BMI after direct reproduction has ceased [27, 28]. Given this, our results are consistent with potential energetic surplus after direct reproduction has ceased that lead to elevated lipid biomarkers after the transition to menopause. Our results suggest that increased lipids following menopause may be a human universal across all populations. After direct reproduction declines or ceases, available lipids contribute to increased fat storage, as has been seen in postmenopausal Tsimane women [27], post-menopausal industrial populations [65], and even various mammals [66]. This lipid availability also produces increasingly atherosclerotic lipid profiles. At later ages (by the second decade after menopause), we see declines in nearly all lipids. These changes have been observed across populations and are likely due to the increased costs of somatic maintenance later in life and the associated changes in body mass (e.g. sarcopenia) [67].

Our results also show a strong secular trend. The effect of menopause on Tsimane lipid levels is roughly equivalent of a 5–7 kg/m^2^ increase in BMI, or the equivalent of aging 5–8 years. In addition, we found that menopause status significantly predicts higher odds of clinically high levels of non-HDL, total cholesterol, and triglycerides. VIFs under 2.5 for all variables in all models suggests limited collinearity; age and menopause appear to exert independent effects on lipid profiles. Overall, our results suggest that while increases in total cholesterol, LDL cholesterol, non-HDL cholesterol, triglycerides, and Apo-B post-menopause appear to be a human universal, the magnitude of increase may vary dramatically between populations depending on their pre-menopausal cardiovascular risk.

While our study did not find significant associations between HDL and menopause status, previous studies have reported mixed findings, with some showing increases [21, 64], others decreases [58, 61], and some reporting no association between HDL and menopause [21, 57, 60, 62, 63]. These inconsistencies highlight the need for further research to examine HDL’s relationship to the menopause transition.

### Limitations

The Apo-B sample was a relatively small subsample of women (n = 361) aged 40–91 and mainly cross-sectional (19.4% longitudinal), limiting its power when compared to our other analyses. We also had a limited sample of Moseten participants (n = 271, aged 33–89) compared to Tsimane participants (n = 850, aged 15–92), potentially limiting our results.

We are unable to measure other advanced lipid metabolism markers such as HDL efflux capacity or LDL particle size under field conditions. Previous studies [21] found decreased LDL particle size following the transition to menopause, contributing to an increasingly atherosclerotic lipid profile. Other studies have reported that total HDL and HDL_3_ declined following menopause but did not find significance for HDL_2_, indicating that remodeled particle size and efflux capacity may contribute to altered lipid profiles after the transition to menopause [58].

## CONCLUSIONS

Despite dramatically lower levels of cardiovascular disease than observed in industrialized populations [33, 35], we find increases in five cardiovascular disease risk biomarkers following the menopause transition. These post-menopausal lipid increases reflect similar trends to what has been reported in industrialized populations, though there is a high degree of cross-population variation in the magnitude of these changes. In summary, the cessation of direct reproduction appears to result in higher lipid levels regardless of environment (subsistence vs. industrialized), suggesting that this may be a universal human pattern shaped by our relatively rare life history strategy of an extended post-reproductive lifespan.

## Supplementary Material

SI_eoaf020

## Data Availability

Individual-level data are stored in the THLHP Data Repository and are available through restricted access for ethical reasons. THLHP’s highest priority is the safeguarding of human subjects and minimization of risk to study participants. The THLHP adheres to the ‘CARE Principles for Indigenous Data Governance’ (Collective Benefit, Authority to Control, Responsibility, and Ethics), which assure that the Tsimane (i) have sovereignty over how data are shared, (ii) are the primary gatekeepers determining ethical use, (iii) are actively engaged in data generation, and (iv) derive benefit from data generated and shared for use whenever possible. The THLHP is also committed to the ‘FAIR Guiding Principles for scientific data management and stewardship’ (Findable, Accessible, Interoperable, Reusable). Requests for individual-level data should take the form of an application that details the uses of the data, the research questions to be addressed, procedures that will be used for data security and individual privacy, potential benefits to the study communities, and procedures for assessing and minimizing stigmatizing interpretations of the research results (see the following webpage for links to the data sharing policy and data request forms: https://tsimane.anth.ucsb.edu/data.html). Requests for individual-level data will require institutional IRB approval (even if exempt) and will be reviewed by an Advisory Council composed of Tsimane community leaders, community members, Bolivian scientists, and the THLHP leadership. The study authors and the THLHP leadership are committed to open science and are available to assist interested investigators in preparing data access requests.
