# Supplementary material for: Higher blood lipid levels after the transition to menopause in two forager-horticulturalist populations
Source: Evol Med Public Health. 2025 Jul 20;13(1):201–14. doi: 10.1093/emph/eoaf020 (PMC12409787; doi:10.1093/emph/eoaf020)
Supplement: SI_eoaf020 [file si_eoaf020.docx]

SUPPLEMENTAL METHODS

**Statistical Methods: Cohort Effects**

Tsimane and Moseten participants were separated into pre- and post-menopause cohorts and binned into 5-year age groups: 40-45, 45-50, 50-55, 55-60. We compared mean values of HDL, LDL, total cholesterol, triglycerides, and Apo-B between pre- and post-menopause individuals in each age- and population-matched cohort using Welch’s t-tests. There were insufficient pre- or post-menopause individuals to measure differences in Apo-B in all cohorts except Tsimane age 45-50 and Tsimane age 55-60. There were insufficient pre- or post-menopause individuals to obtain measures for Moseten age 50-55 and 55-60 (see Tables S1 and S2).

**Statistical Methods: Sensitivity Analysis**

We used a Generalized Estimating Equation (GEE) to test mean differences for all biomarkers between individuals that self-reported age at menopause and those assigned menopause status (at age 50). Normalcy of variables was assessed using a Shapiro-Wilk test (W≥0.95 was considered normal distribution) and all variables except triglycerides exhibited a normal distribution. The GEE model was modified for triglycerides to use a Gamma distribution with a log link function (see Table S3).

**Statistical Methods: Clinically High Odds Ratios**

We used logistic regressions to test if post-menopause status predicted clinically high values for HDL (80 mg/dL), LDL (160 mg/dL), non-HDL (160 mg/dL), total cholesterol (240 mg/dL), and triglycerides (200 mg/dL). There were insufficient pre- or post-menopause individuals to measure differences in Apo-B.

SUPPLEMENTAL RESULTS

**Cohort Effects**

We conducted age- and population-matched comparisons for HDL, LDL, total cholesterol, triglycerides, and Apo-B and compared mean values between pre- and post-menopausal women. Among Tsimane women aged 40-45, 45-50, 50-55, and 55-60 we found no significant differences for any measures. We found significant differences in HDL, LDL, and triglycerides in Moseten women aged 40-45, but not in those aged 45-50. All other Moseten cohorts lacked sufficient pre- or post-menopausal women for comparison. As we controlled for age and year in our main model, these results may indicate that these age group cohorts lack the power to detect subtle changes due to their reduced sample size.

| **Table S1: Sensitivity Analysis T-Test Results for Self-Report vs. Assigned Menopause Tsimane Women** | | | | | |
| --- | --- | --- | --- | --- | --- |
|  |  |  |  |  |  |
| **Age** | **Variable** | **T-Statistic** | **95% CI** | **df** | **P Value** |
| **40-45** | HDL | 0.926 | -5.473, 10.290 | 3.288 | 0.417 |
|  | LDL | -1.743 | -46.196, 12.482 | 3.282 | 0.172 |
|  | Total cholesterol | -1.430 | -52.527, 16.331 | 4.218 | 0.222 |
|  | Triglycerides | -1.487 | -121.181, 35.955 | 4.135 | 0.209 |
|  | Apo-B | * |  |  |  |
| **45-50** | HDL | -0.040 | -2.546, 2.447 | 52.200 | 0.969 |
|  | LDL | 1.327 | -3.150, 15.460 | 52.45 | 0.190 |
|  | Total cholesterol | 1.410 | -2.583, 15.065 | 71.985 | 0.163 |
|  | Triglycerides | 0.153 | -11.295, 13.169 | 77.075 | 0.879 |
|  | Apo-B | 0.278 | -5.624, 5.895 | 1.031 | 0.826 |
| **50-55** | HDL | -0.817 | -7.778, 3.664 | 8.760 | 0.436 |
|  | LDL | 1.020 | -11.968, 32.080 | 9.799 | 0.332 |
|  | Total cholesterol | 0.330 | -17.650, 23.777 | 9.921 | 0.748 |
|  | Triglycerides | -0.216 | -24.927, 20.465 | 11.131 | 0.833 |
|  | Apo-B | * |  |  |  |
| **55-60** | HDL | 1.11 | -2.042, 6.400 | 13.629 | 0.286 |
|  | LDL | -0.024 | -29.990, 29.328 | 11.919 | 0.981 |
|  | Total cholesterol | 0.018 | -24.686, 25.087 | 12.228 | 0.986 |
|  | Triglycerides | -1.563 | -49.524, 7.915 | 13.136 | 0.142 |
|  | Apo-B | -1.321 | -0.671, 0.187 | 7.294 | 0.226 |
| **60-65** | HDL | * |  |  |  |
|  | LDL | * |  |  |  |
|  | Total cholesterol | * |  |  |  |
|  | Triglycerides | * |  |  |  |
|  | Apo-B | * |  |  |  |

* indicates NA due to insufficient pre- or post-menopause sample

| **Table S2: Sensitivity Analysis T-Test Results for Self-Report vs. Assigned Menopause Moseten Women** | | | | | |
| --- | --- | --- | --- | --- | --- |
|  |  |  |  |  |  |
| **Age** | **Variable** | **T-Statistic** | **95% CI** | **df** | **P Value** |
| **40-45** | HDL | 2.895 | 1.005, 5.596 | 45.343 | 0.006 |
|  | LDL | 3.153 | 6.302, 30.977 | 19.826 | 0.005 |
|  | Total cholesterol | 2.655 | -0.362, 44.598 | 4.314 | 0.052 |
|  | Triglycerides | 2.456 | 2.494, 59.104 | 9.132 | 0.036 |
|  | Apo-B | * |  |  |  |
| **45-50** | HDL | 0.584 | -2.424, 4.404 | 45.755 | 0.562 |
|  | LDL | -1.198 | -32.061, 8.194 | 39.832 | 0.238 |
|  | Total cholesterol | -1.891 | -49.683, 1.984 | 27.904 | 0.069 |
|  | Triglycerides | -1.086 | -78.356, 201.733 | 33.303 | 0.286 |
|  | Apo-B | * |  |  |  |
| **50-55** | HDL | * |  |  |  |
|  | LDL | * |  |  |  |
|  | Total cholesterol | * |  |  |  |
|  | Triglycerides | * |  |  |  |
|  | Apo-B | * |  |  |  |
| **55-60** | HDL | * |  |  |  |
|  | LDL | * |  |  |  |
|  | Total cholesterol | * |  |  |  |
|  | Triglycerides | * |  |  |  |
|  | Apo-B | * |  |  |  |
| **60-65** | HDL | * |  |  |  |
|  | LDL | * |  |  |  |
|  | Total cholesterol | * |  |  |  |
|  | Triglycerides | * |  |  |  |
|  | Apo-B | * |  |  |  |

* indicates NA due to insufficient pre- or post-menopause sample

| **Table S3: Generalized Estimating Equation (GEE) Sensitivity Analyses values for LDL, HDL, total cholesterol, triglycerides, and Apo-B** | | | | |  |
| --- | --- | --- | --- | --- | --- |
|  |  |  |  |  |  |
|  | **Term** | **Estimate** | **Standard Error** | **Wald Statistic** | **P Value** |
| Total cholesterol | \| Intercept \| \| --- \| | 157.884 | 3.000 | 2770.337 | <2e-16 |
|  | Group | -3.414 | 1.971 | 3.002 | 0.0832 |
| HDL | Intercept | 34.256 | 0.662 | 2674.4 | <2e-16 |
|  | Group | 1.762 | 0.449 | 15.4 | 8.7e-05 |
| LDL | Intercept | 99.859 | 2.920 | 1169.52 | <2e-16 |
|  | Group | -0.935 | 1.962 | 0.23 | 0.63 |
| Triglycerides | Intercept | 5.045 | 0.042 | 14205.0 | <2e-16 |
|  | Group | -0.145 | 0.0281 | 26.5 | 2.6e-07 |
| Apo-B | Intercept | 13.532 | 0.081 | 27832.33 | <2e-16 |
|  | Group | 0.052 | 0.050 | 1.07 | 0.3 |

| **Table S3: Generalized Estimating Equation (GEE) Sensitivity Analyses values for LDL, HDL, total cholesterol, triglycerides, and Apo-B** | | | | |  |
| --- | --- | --- | --- | --- | --- |
|  |  |  |  |  |  |
|  | **Term** | **Estimate** | **Standard Error** | **Wald Statistic** | **P Value** |
| Total cholesterol | \| Intercept \| \| --- \| | 157.884 | 3.000 | 2770.337 | <2e-16 |
|  | Group | -3.414 | 1.971 | 3.002 | 0.0832 |
| HDL | Intercept | 34.256 | 0.662 | 2674.4 | <2e-16 |
|  | Group | 1.762 | 0.449 | 15.4 | 8.7e-05 |
| LDL | Intercept | 99.859 | 2.920 | 1169.52 | <2e-16 |
|  | Group | -0.935 | 1.962 | 0.23 | 0.63 |
| Triglycerides | Intercept | 5.045 | 0.042 | 14205.0 | <2e-16 |
|  | Group | -0.145 | 0.0281 | 26.5 | 2.6e-07 |
| Apo-B | Intercept | 13.532 | 0.081 | 27832.33 | <2e-16 |
|  | Group | 0.052 | 0.050 | 1.07 | 0.3 |

| **Table S3: Generalized Estimating Equation (GEE) Sensitivity Analyses values for LDL, HDL, total cholesterol, triglycerides, and Apo-B** | | | | |  |
| --- | --- | --- | --- | --- | --- |
|  |  |  |  |  |  |
|  | **Term** | **Estimate** | **Standard Error** | **Wald Statistic** | **P Value** |
| Total cholesterol | \| Intercept \| \| --- \| | 157.884 | 3.000 | 2770.337 | <2e-16 |
|  | Group | -3.414 | 1.971 | 3.002 | 0.0832 |
| HDL | Intercept | 34.256 | 0.662 | 2674.4 | <2e-16 |
|  | Group | 1.762 | 0.449 | 15.4 | 8.7e-05 |
| LDL | Intercept | 99.859 | 2.920 | 1169.52 | <2e-16 |
|  | Group | -0.935 | 1.962 | 0.23 | 0.63 |
| Triglycerides | Intercept | 5.045 | 0.042 | 14205.0 | <2e-16 |
|  | Group | -0.145 | 0.0281 | 26.5 | 2.6e-07 |
| Apo-B | Intercept | 13.532 | 0.081 | 27832.33 | <2e-16 |
|  | Group | 0.052 | 0.050 | 1.07 | 0.3 |

| **Table S4: Linear Regressions comparing pre-menopause, post-menopause, and percent change U.S./U.K. and Tsimane/Moseten total cholesterol, HDL, LDL, non-HDL, and triglycerides** | | | | |
| --- | --- | --- | --- | --- |
| **Pre-menopause** | **Term** | **β** | **Standard Error** | **P Value** |
| Total cholesterol | \| Intercept \| \| --- \| | 180.65 | 7.88 | 2.1e-05 |
|  | Group | -33.65 | 13.65 | 0.069 |
| HDL | Intercept | 62.32 | 2.48 | 1.5e-05 |
|  | Group | -26.38 | 4.30 | 0.0036 |
| LDL | Intercept | 108.92 | 3.70 | 8.4e-07 |
|  | Group | -13.77 | 6.91 | 0.1 |
| Non-HDL | Intercept | 104.25 | 8.05 | 0.0059 |
|  | Group | 6.80 | 11.39 | 0.6109 |
| Triglycerides | Intercept | 78.1 | 10.2 | 0.0006 |
|  | Group | 51.4 | 19.0 | 0.0428 |
| **Post-menopause**  Total cholesterol | Intercept | 212.00 | 48.09 | 1.1e-06 |
|  | Group | -53.50 | -7.01 | 0.0022 |
| HDL | Intercept | 60.00 | 1.81 | 4.9e-06 |
|  | Group | -23.90 | 3.13 | 0.0016 |
| LDL | Intercept | 128.78 | 4.06 | 5.8e-07 |
|  | Group | -27.03 | 7.59 | 0.016 |
| Non-HDL | Intercept | 145.8 | 10.1 | 0.0048 |
|  | Group | -23.4 | 14.3 | 0.2421 |
| Triglycerides | Intercept | 96.4 | 11.0 | 0.00032 |
|  | Group | 45.1 | 20.5 | 0.07924 |
| **Percent Change** |  |  |  |  |
| Total cholesterol | \| Intercept \| \| --- \| | 18.32 | 6.12 | 0.04 |
|  | Group | -12.02 | 10.61 | 0.32 |
| HDL | Intercept | -0.025 | 2.293 | 0.99 |
|  | Group | 0.425 | 3.972 | 0.92 |
| LDL | Intercept | 18.88 | 5.84 | 0.023 |
|  | Group | -11.73 | 10.92 | 0.332 |
| Non-HDL | Intercept | 41.8 | 15.1 | 0.11 |
|  | Group | -31.8 | 21.4 | 0.28 |
| Triglycerides | Intercept | 25.1 | 8.0 | 0.026 |
|  | Group | -15.5 | 15.0 | 0.348 |
